# QSCNAS: A platform for quorum sensing and quenching bacteria analysis in global wastewater treatment plants

**DOI:** 10.1002/imt2.70026

**Published:** 2025-04-07

**Authors:** Yong‐Chao Wang, Sen Wang, Ya‐Hui Lv, Can Wang

**Affiliations:** ^1^ School of Environmental Science and Engineering Tianjin University Tianjin China; ^2^ Tianjin Key Lab of Indoor Air Environmental Quality Control Tianjin China

## Abstract

This study identifies the potential quorum sensing (QS) bacteria in wastewater treatment plants (WWTPs) and constructs a QS communication network through the establishment of a local QS bacterial database with six languages and the analysis of over 1000 activated sludge microbiome samples collected from 269 WWTPs. The results not only advance the understanding of bacterial communication in WWTPs but also provide a valuable tool for developing regulatory strategies to optimize the functionality of these vital ecosystems.

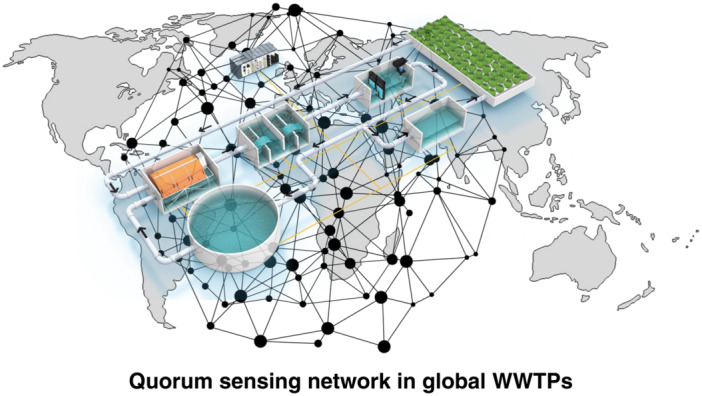

To the Editor,

The application of sociobiology to microbiology has provided valuable insights into the social behaviors and information transmission within microbial communities [1, 2]. It is now acknowledged that quorum sensing (QS), which relies on signaling molecular transduction, governs the expression of specific genes and the regulation of population behavior [3, 4, 5]. Nevertheless, the specificity of microbial communities and the diversity of QS signal types across various habitats present significant challenges in deciphering and governing the complex communication systems of the microbial world [1, 6, 7]. The creation of specialized key knowledge maps for various ecosystems has increasingly been recognized as an effective strategy to enhance the application of QS therapy.

As the world's largest biotechnology by volume, the function of activated sludge (AS) systems in wastewater treatment plants (WWTPs) is directly reflected in its ability to remove pollutants [8, 9]. Therefore, the influence of QS on microbial population behavior can be further reflected in AS. Although the significance of QS behaviors within the microbial ecosystem emerged in the interest of engineers [10], it remains a huge challenge to link the QS signals, bacteria relative abundance, and ecosystem functions. For example, although the co‐occurrence and interaction of multiple QS signals have been documented in anaerobic ammonium oxidation (anammox) system and biofilters, the development of targeted control strategies for process enhancement is still in the early stage [4, 11]. To help understand the effects of QS behavior on microbial interactions and functions in WWTPs, we developed a global analysis of QS and quorum quenching (QQ) bacteria in WWTPs based on AS microbiome samples collected from 269 WWTPs and conducted an online platform named QS communication network in AS (QSCNAS) to explore the language‐based interactions within the microbial community. The potential pathways of QS communication influencing ecosystem functions were uncovered by associating pollutant removal efficiency with the QS network. The results offered potential guiding strategies for enhancing the performance of WWTPs based on bacterial interaction regulation.

## RESULTS AND DISCUSSION

### Overview of QSCNAS

**Figure 1:**
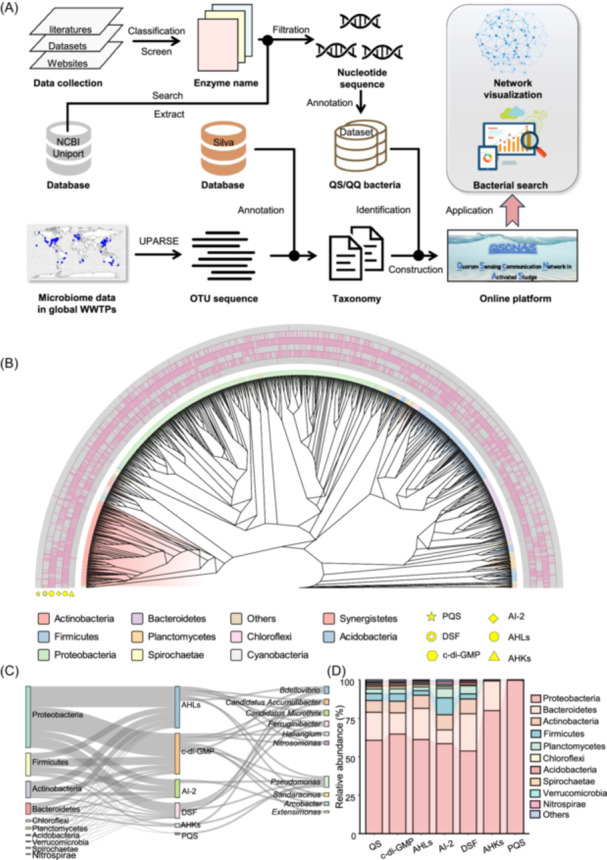
Construction of quorum sensing communication network in WWTPs. (A) Enzyme names related to QS and QQ were collected from published literature, databases, and websites, and then the corresponding gene sequence information was extracted from the NCBI database and further annotated to bacterial taxonomy to obtain a local QS/QQ bacterial database. Subsequently, the 1186 samples collected from global WWTPs were analyzed to obtain the microbiome information, and QS/QQ bacteria were identified using the local QS/QQ bacterial database. (B) Phylogenetic tree of the six types of QS bacteria and their phylum distribution. The heatmap of the outer ring indicates the presence or absence of QS function in the bacterium. Symbol types represent different types of QS languages. (C) Basic taxonomic composition of the QS bacteria at the phylum (left) and genus (right) level. Only the top 10 phyla and genera are shown in the figure. (D) The relative abundance of the top 10 phyla of the six types of QS bacteria. The first column represents the relative abundance of the total QS bacteria. The abundance of each phylum was normalized using the total abundance of QS‐associated bacterial populations. AHKs, *α*‐hydroxyketones; AHLs, acyl‐homoserine lactones; AI‐2, autoinducers‐2; c‐di‐GMP, bis‐(3'‐5')‐cyclic dimeric guanosine monophosphate; DSF, diffusion‐signaling factors; OTU, operational taxonomic unit; PQS, quinolone signal; QQ, quorum quenching; QS, quorum sensing; WWTPs, wastewater treatment plants.

An online platform named QSCNAS was developed to explore QS‐mediated communication and interactions within WWTPs by constructing a local QS/QQ database and analyzing AS microbiome data from global WWTPs (Figure 1A). Based on the mechanisms of bacterial communication and quenching, we gathered 23 gene subtypes involved in signal molecule synthesis, acceptance, and quenching (Table S1). These were then assigned to their respective taxonomies using MEGAN software to create the local QS/QQ database. To mitigate the deviations in the sequencing data across studies, such as sequencing primers and sequencing depths, we utilized a comprehensive global AS microbiome sample data set compiled by the Global Water Microbiome Consortium (GWMC) [12]. This data set includes samples from 269 WWTPs across 23 countries, all sequenced uniformly on the same MiSeq instrument. The majority of samples (over 95%) were sequenced multiple times to ensure consistency in sequencing reads (approximately 30,000). These analyses allowed us to identify potential QS and QQ bacteria in WWTPs and enabled the creation of an online platform dedicated to the study of QS interactions in AS (http://www.qscnas.cn/).

### The distribution of various QS languages in WWTPs

The distribution of QS language is depicted by constructing a phylogenetic tree of QS bacteria in WWTPs (Figure 1B). Intuitively, it can be observed that bacteria related to bis‐(3'‐5')‐cyclic dimeric guanosine monophosphate (c‐di‐GMP) and acyl‐homoserine lactones (AHLs) dominate the AS bacterial communication system, with 85% and 75% of QS bacteria performing functions related to these two signaling molecules, respectively (Figure S1). The proportion of bacteria associated with diffusion‐signaling factors (DSF) and autoinducers−2 (AI‐2) signaling systems was nearly 40%. Bacteria related to *α*‐hydroxyketones (AHKs) and quinolone signal (PQS) account for the least proportion. In contrast, bacteria that can degrade AHL signaling molecules became the dominant species within the QQ communities, which account for nearly 80% (Figure S2). This may be duo to the high proportion of AHL in QQ analysis. Although it has been reported that the concentration of c‐di‐GMP is maintained in a state of homeostasis within bacteria [6, 11], the number of bacteria possessing the phosphodiesterase (PDE; degradation enzyme) domain is significantly lower than the number with the diguanylate cyclase (DGC; synthesis enzyme) domain. This might be due to the fact a large number of DGC effectors in bacteria, which could perceive the c‐di‐GMP concentration and increased stability of c‐di‐GMP signaling networks by maintaining c‐di‐GMP levels in a defined concentration window [13].

**Figure 2:**
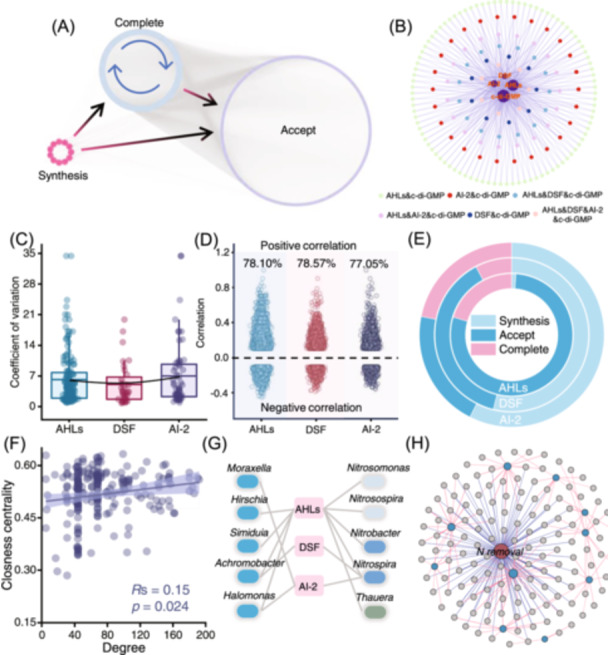
Analysis of quorum sensing (QS) communication networks in WWTPs. (A) AHL‐type QS bacteria‐directed communication network is established from AHL signal molecule synthesis to acceptance. (B) Multisignal bacterial identification network. This network is constructed using the four main types of signaling molecules and bacteria containing more than two related functions. The colors from the outside to the inside indicate the related bacteria of different multi‐level signaling regulatory pathways. The purple circles in the middle (from large to small) represent c‐di‐GMP, AHLs, AI‐2, and DSF signal molecules, respectively. (C) The coefficient of variation analysis shows the difference between the three types of QS completers in WWTPs. (D) The proportion of positive and negative correlations in bacterial abundance co‐occurrence networks in three main QS languages. (E) Proportion of keystone bacteria in synthetic, accepting, and complete bacteria in the three main QS language bacterial co‐occurrence networks. Bacteria that contain both synthetic and acceptance functions are defined as complete bacteria. (F) Linear fittings between the closeness centrality in the abundance co‐occurrence network and degree of bacteria in QS interspecific communication network. (G) Diagram of potential communication languages between QS bacteria that are positively correlated with removal performance and nitrogen removal functional bacteria. (H) The co‐occurrence network revealed the effect of QS bacteria on nitrogen removal. The red node indicated removal performance. The blue and gray nodes represent the functional QS bacteria and other significantly correlated QS bacteria, respectively. The edges represent positive correlations. The blue edges indicate the direct effect of QS bacteria on removal performance, and the red edges indicate that these QS bacteria may affect removal performance by influencing functional bacteria. AHLs, acyl‐homoserine lactones; AI‐2, autoinducers‐2; c‐di‐GMP, bis‐(3'‐5')‐cyclic dimeric guanosine monophosphate; DSF, diffusion‐signaling factors.

Taxonomic analysis demonstrated that Proteobacteria, representing over 50% of the relative abundance across various communication systems, encompassed nearly all functional capabilities related to both signal production and quenching activities (Figure 1C and Figure S2). Besides, the major phyla found in WWTPs, including Bacteroidetes, Firmicutes, and Actinobacteria, were also present in high relative abundance within the QS and QQ communities (Figure 1D). Meanwhile, some certain functionally important bacteria, such as the phosphorus‐accumulating bacterium *Candidatus* Accumulibacter and the filamentous sludge‐bulking bacterium *Candidatus* microthrix, are not only abundant in global WWTPs but also associated with multiple QS signaling languages (Figure 1B). Specifically, we found that over half of the QS bacteria can share more than two types of QS languages (Figure S3). For example, the majority of the top 10 microbial genera in terms of relative abundance within WWTPs could share c‐di‐GMP and AHLs (Figure S4). More than 100 genera were found to share more than four QS languages (Figure S3). Such complex cross‐talk between QS languages in AS provided opportunities to facilitate multi‐level intraspecific QS regulation. Therefore, four main signals, including the c‐di‐GMP, AHLs, DSF, and AI‐2, were selected for further analysis (Figure 2A,B).

### Comparison of diverse QS languages in WWTPs

Given the complexity of QS languages in AS ecosystems, we developed three QS‐associated bacterial networks to elucidate signal‐specific communication patterns (Figure 2A and Figure S5). According to the type of QS signal synthesizing and accepting, the QS language‐related bacteria were divided into three categories: synthesizer (only contains the synthesis function), acceptor (only contains the accept function), and completer (contains both the synthesis and accept functions). Interestingly, the number of acceptors in AHLs‐type QS bacteria is much greater than that of synthesizers, which is in stark contrast to DSF and AI‐2‐type QS bacteria (Figures S5 and S6). These suggested that AHL demand within the microbial community may exceed its synthetic capacity, potentially intensifying competition among AHL acceptors [1, 3, 14]. Additionally, bacteria classified as completers exhibited high relative abundance and frequency of occurrence, along with low coefficient of variation across all three QS communication networks, as shown in Figures S5–S7. Meanwhile, DSF‐related completers are superior to those of AHLs and AI‐2 in abundance, occurrence frequency, and coefficient of variation (Figure 2C and Figure S7), which further demonstrates their importance and contribution to creating social benefits. Co‐occurrence networks in the three QS languages showed bacteria involved in QS communication had strong positive correlations (77.94% ± 0.62%) (Figure 2D and Figure S8). These indicated the importance of QS in mediating cooperative behavior in microbial communities. In addition, a considerable number of keystone nodes (26.03% ± 10.77%) were identified across the three separate co‐occurrence networks (Figure S9). The distribution of the keystone nodes among the three QS categories corresponds consistently with the QS communication networks (Figure 2E).

### Multisignal bacterial identification and interspecific communication network analysis

The bacterial communication networks were further analyzed through single‐mode network characterization to identify bacteria involved in multi‐level regulation and to investigate potential interspecific communication relationships (Figure 2B and Figure S10). To identify the bacteria that contain multi‐level signaling regulatory pathways, we constructed a hypothesized network using the four main types of signaling molecules and bacteria containing more than two related functions (Figure 2B). Briefly, the ubiquitous presence of c‐di‐GMP across all network nodes containing multiple potential signaling pathways, indicating the importance of c‐di‐GMP in complex multi‐level signaling path regulation. Additionally, the network also identified certain microbial genera that might be involved in multiple QS regulatory pathways. For instance, we found that *Salmonella* appeared to be regulated by multiple signaling molecules, including AHLs, AI‐2, and c‐di‐GMP (Figure S11), which was confirmed in previous studies [15]. Besides, we also found that the ecological impact of these multilevel intraspecific QS bacteria did not seem to be directly related to the number of QS signals involved (Figure S12). Although the genus *Succinivibrio* only contains two signals (c‐di‐GMP and AHLs), it exhibited a high keystoneness within the community (Figure S11).

Interspecific communication was also evidenced to be a strategy for microbial communities to respond to environmental stress (Figure S13) [12]. Therefore, a directed multilevel interspecific communication network was constructed by linking the potential interspecific QS language (AHLs, DSF, and AI‐2) related bacteria (Figure S10). Among them, 46.71% of the relationships are composed of parallel edges of DSF and AHLs, followed by AI‐2 and AHLs, suggesting complex QS language communication in AS communities. Meanwhile, we also constructed the co‐occurrence network and observed a high degree (78%) of cooperative behavior among these bacteria (Figure S14). In addition, it was astonishing to find that the majority of genera (95%) serve as keystone nodes, including the top 20 genera in keystoneness prediction (Figures S10 and S14). Especially, the network parameters of these multilevel interspecific communication bacteria in QS networks showed a significant positive correlation (*p* < 0.05) with their parameters in the co‐occurrence network, as well as with their relative abundance and coefficient of variation in global WWTPs (Figure 2F and Figure S15). Meanwhile, QS‐mediated bacterial communication, including both interspecific and intraspecific interactions, exhibited significantly higher correlations (*p* < 0.05) with performance metrics (such as nitrogen removal) than the overall microbial community (Figure S12).

### Linkage between QS behavior and the functions of the WWTPs

To reveal the influence of QS communication network on the functions of the WWTPs, we explored the connections between QS bacteria and function bacteria and the removal performance of typical pollutants, such as nitrogen. We initially searched the functional bacteria associated with nitrogen removal based on the MIDAS4 database and then constructed the nitrogen removal communication network by connecting functional bacteria with QS bacteria with potential language connections (Figure S16). The results showed that *Nitrospira* and *Rhodobacter* presented relatively high QS connections in the community, which might act as the main bridges in QS communication networks to facilitate function achievements. Meanwhile, QS bacteria in the nitrogen removal network displayed strong synergy with functional bacteria, with over 70% of co‐occurrence edges showing positive correlations (Figure S16). In addition, it was surprising to find that the abundance of these QS bacteria also showed a clear positive correlation (76.56%) with performance, indicating the critical role of the functional bacteria in linking removal performance and QS behaviors. The construction of the nitrogen removal network suggested that the QS communication bacteria might promote nitrogen removal performance through direct or indirect QS signals (Figure 2G,H). For instance, the top five bacteria with the highest correlations in terms of nitrogen removal could present direct links with ammonia‐oxidizing bacteria, nitrifying bacteria, and denitrifying bacteria using QS signals (Figure 2G). Besides, we also found the effect of environmental variables on QS communities, which may further affect nitrogen removal performance (Figure S17). Specifically, the QS community composition was closely correlated with the F/M (the ratio of organic matter to microorganisms) and sludge volume index (Mantel's *r* > 0.2, *p* < 0.01). Temperature and influent nitrogen concentration were found as the main factors that induced strong positive and direct effects on QS community composition (standardized path coefficient, *β* > 0.50, *p* < 0.001).

## CONCLUSION

In summary, this study developed the QSCNAS to analyze the links between QS signals, bacteria relative abundance, and the functions of WWTPs. Compared with the bacteria that only contain signal synthesis or acceptance, the bacteria that fully possess both functions have higher relative abundance and occurrence frequency and lower coefficient of variation. The construction of intraspecific and interspecific multi‐level QS communication networks shed light on the complexity and interplay of the QS language. The establishment of potential function QS networks revealed the influence mechanisms of bacterial communication on the pollutant removal process. Overall, the results highlighted the importance of QS communication in microbial interactions, which further provided potential guidance for QS therapy in wastewater treatment processes.

## AUTHOR CONTRIBUTIONS

**Yong‐Chao Wang:** Conceptualization; investigation; writing—original draft; methodology; validation; data curation; formal analysis. **Sen Wang:** Investigation; methodology; software; visualization; data curation. **Ya‐Hui Lv:** Investigation; conceptualization; methodology; formal analysis; resources. **Can Wang:** Writing—review & editing; project administration; supervision; funding acquisition, investigation; formal analysis; data curation. All authors have read the final manuscript and approved it for publication.

## CONFLICT OF INTEREST STATEMENT

The authors declare no conflicts of interest.

## ETHICS STATEMENT

No animals or humans were involved in this study.

## Supporting information


**Figure S1.** The number of genera associated with different signaling molecules in AS system.
**Figure S2.** Distribution of QQ bacteria in WWTPs.
**Figure S3.** The analysis of QS crosstalk in WWTPs.
**Figure S4.** Diagram of potential communication languages between the top 10 genera in relative abundance in WWTPs and the diverse QS languages.
**Figure S5.** Analysis of the AI−2 type QS bacteria in WWTPs.
**Figure S6.** Analysis of the DSF type QS bacteria in WWTPs.
**Figure S7.** Comparison of various types of QS bacteria in WWTPs.
**Figure S8.** Co‐occurrence networks of AHLs, DSF, and AI−2 types of QS bacteria.
**Figure S9.** Identification of keystone bacteria in the AHLs, AI−2, and DSF types of QS bacterial co‐occurrence networks.
**Figure S10.** Bacterial interspecific communication network construction.
**Figure S11.** Analysis of intraspecific communication networks in WWTPs.
**Figure S12.** Analysis of various QS bacteria in influencing the functions of WWTPs.
**Figure S13.** Schematic diagram of microbial QS interspecific communication.
**Figure S14.** Co‐occurrence network of the interspecific QS bacteria.
**Figure S15.** Linear fittings between the parameters between QS communication network and co‐occurrence network.
**Figure S16.** The linkage between bacterial communication and the functions of WWTPs.
**Figure S17.** The analysis of the impact of operation conditions on QS community.

**Table S1.** Protein names related to synthesis, acceptance, and quenching of signal molecules in different language systems.

## Data Availability

The global microbiome database of activated sludge can be obtained in NCBI with accession number PRJNA509305 (https://www.ncbi.nlm.nih.gov/bioproject/?term=PRJNA509305). The representative sequences of the global activated sludge microbiome are available at the website (http://gwmc.ou.edu/data-disclose.html). More details about the quorum sensing communication network can be found on the online platform (http://www.qscnas.cn). The data and scripts used are saved in GitHub https://github.com/basswilson/Canlab-QSCNAS. Supplementary materials (methods, figures, tables, graphical abstract, slides, videos, Chinese translated version, and update materials) may be found in the online DOI or iMeta Science http://www.imeta.science/.
