# Supplementary material for: QSCNAS: A platform for quorum sensing and quenching bacteria analysis in global wastewater treatment plants
Source: Imeta. 2025 Apr 7;4(3):e70026. doi: 10.1002/imt2.70026 (PMC12130577; doi:10.1002/imt2.70026)
Supplement: Supplementary file 1 — Figure S1. The number of genera associated with different signaling molecules in AS system. Figure S2. Distribution of QQ bacteria in WWTPs. Figure S3. The analysis of QS crosstalk in WWTPs. Figure S4. Diagram of potential communication languages between the top 10 genera in relative abundance in WWTPs and the diverse QS languages. Figure S5. Analysis of the AI−2 type QS bacteria in WWTPs. Figure S6. Analysis of the DSF type QS bacteria in WWTPs. Figure S7. Comparison of various types of QS bacteria in WWTPs. Figure S8. Co‐occurrence networks of AHLs, DSF, and AI−2 types of QS bacteria. Figure S9. Identification of keystone bacteria in the AHLs, AI−2, and DSF types of QS bacterial co‐occurrence networks. Figure S10. Bacterial interspecific communication network construction. Figure S11. Analysis of intraspecific communication networks in WWTPs. Figure S12. Analysis of various QS bacteria in influencing the functions of WWTPs. Figure S13. Schematic diagram of microbial QS interspecific communication. Figure S14. Co‐occurrence network of the interspecific QS bacteria. Figure S15. Linear fittings between the parameters between QS communication network and co‐occurrence network. Figure S16. The linkage between bacterial communication and the functions of WWTPs. Figure S17. The analysis of the impact of operation conditions on QS community. [file IMT2-4-e70026-s002.docx]

**Supporting information to**

**QSCNAS: A platform for quorum sensing and quenching bacteria analysis in global wastewater treatment plants**

**Running title:** Bacterial communication analysis in wastewater treatment plants

Yong-Chao Wang^1,2^, Sen Wang^1,2^, Ya-Hui Lv^1,2^, Can Wang^1,2^*

^1^School of Environmental Science and Engineering, Tianjin University, Tianjin 300072, China

^2^Tianjin Key Lab of Indoor Air Environmental Quality Control, Tianjin 300072, China

*Correspondence: [wangcan@tju.edu.cn](mailto:wangcan@tju.edu.cn) (Can Wang)

**METHOD**

**Construction of QSCNAS in global wastewater treatment plants**

The construction of QSCNAS is divided into two parts, including the establishment of a local QS/QQ database and the analysis global AS microbiome dataset. Firstly, five types of signaling molecules, including AHLs, DSF, AI-2, AHKs, and PQS, were selected through the investigation of existing QS literature, websites, and databases, such as QSDB, BCG, SigMol, Uniport, and QSP [1−3]. Subsequently, 17 QS-related gene subtypes containing synthesis and acceptance of the above six signaling molecules were collected for sequence collection (Table S1). Meanwhile, six corresponding gene subtypes of QQ enzymes, including lactonase, acylase et al., were also collected for the construction of QQ bacterial database (Table S1) [4,5]. Additionally, the universal bacterial secondary messenger c-di-GMP was also selected because it related to bacterial communication. The c-di-GMP degradation enzyme (phosphodiesterase (PDE)) and synthesis enzyme (diguanylate cyclase (DGC)) were collected. According to these gene subtypes, relevant amino acid sequences were retrieved from the Uniport, QSP, National Center for Biotechnology Information (NCBI), and Kyoto Encyclopedia of Genes and Genomes (KEGG) database and filtered. These sequences were then analyzed using Diamond with a non-redundant (NR) protein sequence database to assign taxonomic information, which was further processed by MEGAN (v 6.0) software for the generation of taxonomic profiles [2,3].

To obtain taxonomic information for bacteria in AS, we collected and re-analyzed data on the microbiome from the Global Water Microbiome Consortium (GWMC) [6]. This data set includes 1186 AS samples, as well as their corresponding physical, chemical properties, environmental factors, and pollutant removal performance, which were collected from 269 WWTPs worldwide. These samples were sequenced using the V4 (515F/806R) region and the UPARSE was applied to obtain the operational taxonomic unit (OTU) information at the 97% similarity threshold [7]. To ensure the uniformity of analysis, the sequence number in each sample was rarefied to the same depth (25,600 sequences per sample), which results in 61,448 OTUs for subsequent comparative analyses. Then the taxonomic information in global WWTPs was assigned using the representative sequences based on the Silva v138 database at a 97% similarity level [8]. To ensure accuracy, the taxonomic information from the QS/QQ bacteria and the global WWTPs were compared at the genus level to obtain the potential bacteria in AS [4]. On this basis, the QSCNAS was constructed to analyze the ecological behavior and microbial interaction in WWTPs.

**Communication network construction**

It has been demonstrated that the different microbes can communicate with each other to form cell-cell communication networks using diverse QS languages [9,10]. Therefore, the potential QS communication networks within WWTPs were established by connecting QS signal-synthesizing bacteria with recipient bacteria. Notably, as the intracellular second messenger, the c-di-GMP-related signal was not considered in QS communication networks [4,11,12]. As recognized interspecific signaling molecules, DSF and AI-2-related bacteria are considered to be involved in interspecific communication [1,13]. Meanwhile, recent studies revealed that AHL signaling molecules are also involved in interspecific communication [9,14]. Hence, the interspecific communication network was constructed based on the bacteria associated with these three types of signaling molecules and then visualized in Cytoscape [14]. To verify the role of QS communication network in microbial communities, the co-occurrence network was constructed based on microbial abundance using iNAP and visualized in Gephi (v 0.9.2) [15]. The keystone nodes in the co-occurrence network were identified by calculating the among-module connectivity (Pi) and within-module connectivity (Zi) [16]. Meanwhile, considering the mutual regulation of multiple QS signal molecules within microbes [11,17], bacteria with multiple QS languages were selected to construct QS multi-level regulatory networks to identify the bacteria that contain multi-level signaling regulatory pathways. Linear fitting of QS communication network and co-occurrence network parameters was used to evaluate the effects of QS communication on microbial interactions. A *p*-value less than 0.05 was considered as the significant correlation. The relative abundance, occurrence frequency, and coefficient of variation of diverse QS language-related bacteria were calculated to assess the relative importance of these languages in WWTPs.

**Estimating keystoneness contributions of the QS bacteria in the community**

To elucidate the ecological role of QS bacteria within AS communities, a deep learning framework was utilized to evaluate the keystoneness of QS genera, as described in a previous study [18] (Figure S1). In essence, a mapping function (*φ*) was developed to connect microbial assemblages with their abundance using a presence matrix and an abundance matrix as the training data. Following this, an experiment was conducted to simulate the removal of each genus (*i*) from the presence matrix, thereby creating modified assemblage matrices that represented the community following the hypothetical loss of each genus. These matrices were then input into the trained mapping function (*φ*) to produce corresponding altered abundance matrices. The impact of each genus on the AS community structure was determined by comparing the original and modified abundance matrices, which allowed for the quantification of each genus’s keystoneness contribution across individual AS samples. Additionally, intraspecific and interspecific QS microbial abundance matrices were calculated separately to establish the relative importance rankings. It is important to note that only genera detected in at least 20% of the samples were included in the calculations.

**Assessment of the effects of QS on WWTP functions**

To evaluate the impact of QS behavior on the functionality of WWTPs, we developed QS communication networks by examining the relationships between functional bacteria and their potential QS-communicating counterparts, as well as co-occurrence networks. Specifically, we identified functional bacteria, such as those involved in nitrogen removal, from the MiDAS 4 database, which provides a comprehensive classification of functional bacteria in WWTPs [19]. At the same time, we corresponded and correlated the performance data obtained from GWMC with the sample microbiome information, as described in previous study [6]. Subsequently, we mapped the QS communication network onto the nitrogen removal functional network by identifying bacteria with QS language links to the functional bacteria. Concurrently, we established a co-occurrence network using the abundance matrix of these bacteria to investigate the correlation (positive or negative) between the functional bacteria and the associated QS bacteria [15]. The influence of these bacteria on WWTP performance was assessed using the Spearman’s rank correlation coefficients between bacterial abundance and treatment performance [6]. A *p*-value (adjusted by the Benjamini-Hochberg method) below 0.05 was considered indicative of a significant correlation. The operational functionality of the WWTPs was quantified as the removal rate (g/g biomass/day) of ammonia nitrogen, as previously described by Wu et al. [6].

It is known that operational parameters could influence process performance through their direct impact on microbial community structure and functional populations. Therefore, we analyzed the impact of operational conditions on QS communities through Mantel test (999 permutations) [6]. The direct impact of operational conditions on communities was further revealed by constructing a structural equation model (SEM) with the richness, PC1 value of principal coordinate analysis, and environmental variable parameters. The model was processed using the lavaan R package and we firstly considered with all possible connections and then eliminated nonsignificant connections until all of the exist connections were significant [6]. The corresponding operating conditions in the GWMC microbiome samples, *e.g.*, temperature, pH, influent rate, contaminant concentration, and other factors are included into the analysis.

**REFERENCES**

1. Rajput, Akanksha, Karambir Kaur, Manoj Kumar. 2016. “SigMol: repertoire of quorum sensing signaling molecules in prokaryotes.” *Nucleic Acids Research* 44: D634−D639. <https://doi.org/10.1093/nar/gkv1076>

2. Dai, Chunxiao, Yuanyuan Qu, Weize Wu, Shuzhen Li, Zhuo Chen, Shengyang Lian, Jiawei Jing. 2023. “QSP: An open sequence database for quorum sensing related gene analysis with an automatic annotation pipeline.” *Water Research* 235: 119814. <https://doi.org/10.1016/j.watres.2023.119814>

3. Du, Qing, Quanhua Mu, Guangxue Wu. 2021. “Metagenomic and bioanalytical insights into quorum sensing of methanogens in anaerobic digestion systems with or without the addition of conductive filter.” *Science of the Total Environment* 763: 144509. <https://doi.org/10.1016/j.scitotenv.2020.144509>

4. Tang, Xi, Yongzhao Guo, Bo Jiang, Sitong Liu. 2018. “Metagenomic approaches to understanding bacterial communication during the anammox reactor start-up.” *Water Research* 136: 95−103. <https://doi.org/10.1016/j.watres.2018.02.054>

5. Wang, Yong-Chao, Yu-Ting Lin, Can Wang, Zhen Tong, Xu-Rui Hu, Ya-Hui Lv, Guan-Yu Jiang, et al. 2022. “Microbial community regulation and performance enhancement in gas biofilters by interrupting bacterial communication.” *Microbiome* 10: 150. <https://doi.org/10.1186/s40168-022-01345-5>

6. Wu, Linwei, Daliang Ning, Bing Zhang, Yong Li, Ping Zhang, Xiaoyu Shan, Qiuting Zhang, et al. 2019. “Global diversity and biogeography of bacterial communities in wastewater treatment plants.” *Nature Microbiology* 4: 1183−1195. <https://doi.org/10.1038/s41564-019-0426-5>

7. Edgar, Robert C. 2013. “UPARSE: highly accurate OTU sequences from microbial amplicon reads.” *Nature Methods* 10: 996−998. <https://doi.org/10.1038/nmeth.2604>

8. Quast, Christian, Elmar Pruesse, Pelin Yilmaz, Jan Gerken, Timmy Schweer, Pablo Yarza, Jörg Peplies, Frank Oliver Glöckner. 2013. “The SILVA ribosomal RNA gene database project: improved data processing and web-based tools.” *Nucleic Acids Research* 41: D590−D596. <https://doi.org/10.1093/nar/gks1219>

9. Wu, Shengbo, Jie Feng, Chunjiang Liu, Hao Wu, Zekai Qiu, Jianjun Ge, Shuyang Sun, et al. 2022. “Machine learning aided construction of the quorum sensing communication network for human gut microbiota.” *Nature Communications* 13: 3079. <https://doi.org/10.1038/s41467-022-30741-6>

10. Wellington, Samantha, E. Peter Greenberg. 2019. “Quorum sensing signal selectivity and the potential for interspecies cross talk.” *mBio* 10: e00146-19. <https://doi.org/10.1128/mbio.00146-19>

11. Pan, Juejun, Jianhang Zhou, Xi Tang, Yongzhao Guo, Yunpeng Zhao, Sitong Liu. 2023. “Bacterial communication coordinated behaviors of whole communities to cope with environmental changes.” *Environmental Science & Technology* 57: 4253−4265. <https://doi.org/10.1021/acs.est.2c05780>

12. Wang, Yongchao, Ya-Hui Lv, Yu-Ting Lin, Yi-Mei Xue, Xu-Rui Hu, Guanyu Jiang, Can Wang. 2024. “Improving biofilm formation and bioreactor startup at low temperature through regulating microbial community communication and stability.” *ACS ES&T Water* 4: 2894−2905. <https://doi.org/10.1021/acsestwater.4c00103>

13. Papenfort, Kai, Bonnie L. Bassler. 2016. “Quorum sensing signal–response systems in Gram-negative bacteria.” *Nature Reviews Microbiology* 14: 576−588. <https://doi.org/10.1038/nrmicro.2016.89>

14. Jin, Ying, Wenkang Chen, Jie Hu, Jinfeng Wang, Hongqiang Ren. 2024. “Constructions of quorum sensing signaling network for activated sludge microbial community.” *ISME Communications* 4: ycae018. <https://doi.org/10.1093/ismeco/ycae018>

15. Feng, Kai, Xi Peng, Zheng Zhang, Songsong Gu, Qing He, Wenli Shen, Zhujun Wang, et al. 2022. “iNAP: An integrated network analysis pipeline for microbiome studies.” *iMeta* 1: e13. <https://doi.org/10.1002/imt2.13>

16. Wen, Tao, Penghao Xie, Shengdie Yang, Guoqing Niu, Xiaoyu Liu, Zhexu Ding, Chao Xue, Yong-Xin Liu, Qirong Shen, Jun Yuan. 2022. “ggClusterNet: An R package for microbiome network analysis and modularity-based multiple network layouts.” *iMeta* 1: e32. <https://doi.org/10.1002/imt2.32>

17. Zhang, Lei, Shuyu Li, Xiaozhen Liu, Zhuo Wang, Mei Jiang, Ruiying Wang, Laigong Xie, et al. 2020. “Sensing of autoinducer-2 by functionally distinct receptors in prokaryotes.” *Nature Communications* 11: 5371. <https://doi.org/10.1038/s41467-020-19243-5>

18. Wang, Xu-Wen, Zheng Sun, Huijue Jia, Sebastian Michel-Mata, Marco Tulio Angulo, Lei Dai, Xuesong He, Scott T. Weiss, Yang-Yu Liu. 2024. “Identifying keystone species in microbial communities using deep learning.” *Nature Ecology & Evolution* 8: 22−31. <https://doi.org/10.1038/s41559-023-02250-2>

19. Dueholm, Morten Kam Dahl, Marta Nierychlo, Kasper Skytte Andersen, Vibeke Rudkjøbing, Simon Knutsson, Sonia Arriaga, Rune Bakke, et al. 2022. “MiDAS 4: A global catalogue of full-length 16S rRNA gene sequences and taxonomy for studies of bacterial communities in wastewater treatment plants.” *Nature Communications* 13: 1908. <https://doi.org/10.1038/s41467-022-29438-7>

20. Li, Shuyu, Hengxi Sun, Jianghan Li, Yujiao Zhao, Ruiying Wang, Lei Xu, Chongyi Duan, et al. 2022. “Autoinducer-2 and bile salts induce c-di-GMP synthesis to repress the T3SS via a T3SS chaperone.” *Nature Communications* 13: 6684. <https://doi.org/10.1038/s41467-022-34607-9>

21. Swofford, Charles A., Nele Van Dessel, Neil S. Forbes. 2015. “Quorum-sensing Salmonella selectively trigger protein expression within tumors.” *Proceedings of the National Academy of Sciences of the United States of America* 112: 3457−3462. <https://doi.org/10.1073/pnas.1414558112>


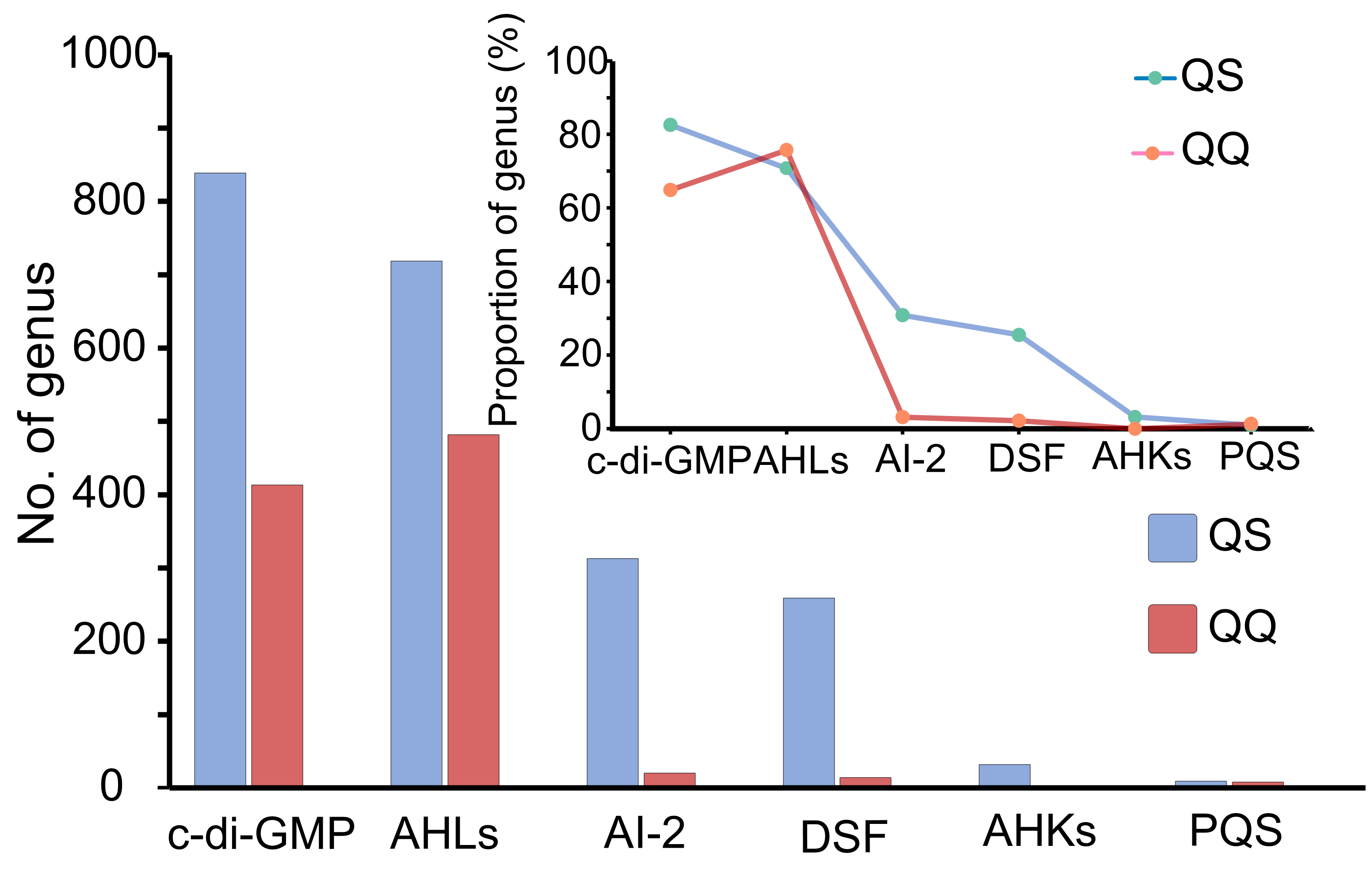


Figure S1 The number of genera associated with different signaling molecules in AS system. The insert represents the proportion of specific QS bacteria in the total QS bacteria community. QS, quorum sensing; QQ, quorum quenching; c-di-GMP, bis-(3’-5’)-cyclic dimeric guanosine monophosphate; AHLs, acyl-homoserine lactones; DSF, diffusion-signaling factors; AI-2, autoinducers-2; AHKs, *α*-hydroxyketones; PQS, quinolone signal.


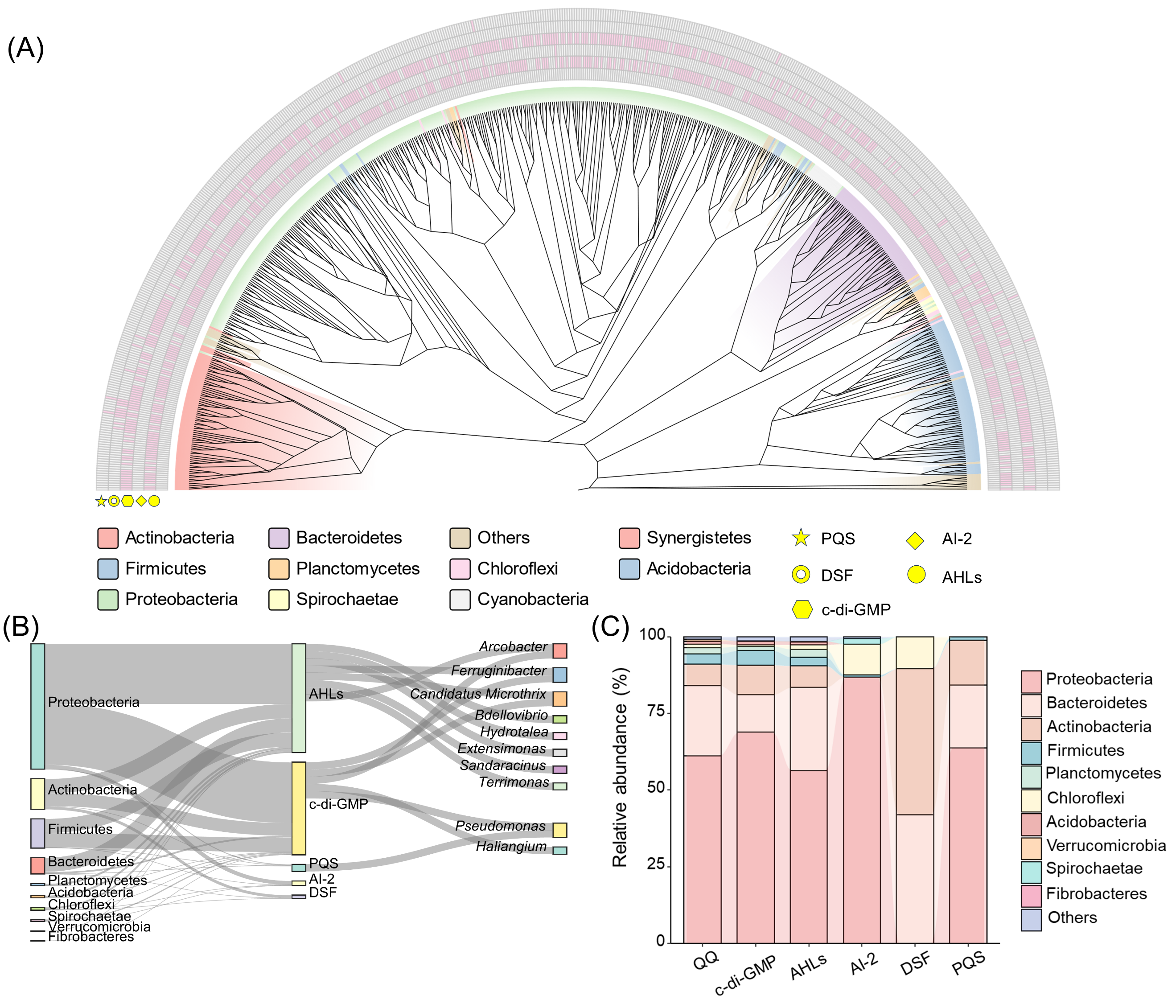
Figure S2 Distribution of QQ bacteria in WWTPs. (A) Phylogenetic tree of the five types of QQ bacteria and their phylum distribution. The heatmap of the outer ring indicates the presence or absence of QQ function in the bacterium. Symbol types represent different types of QQ functions. (B) Basic taxonomic composition of the QQ bacteria at the phylum (left) and genus (right) level. Only the top 10 phyla and genera are shown in the figure. (C) The relative abundance of the top 10 phyla of the five types of QQ bacteria. The first column represents the relative abundance of the total QQ bacteria. QS, quorum sensing; QQ, quorum quenching; c-di-GMP, bis-(3’-5’)-cyclic dimeric guanosine monophosphate; AHLs, acyl-homoserine lactones; DSF, diffusion-signaling factors; AI-2, autoinducers-2; AHKs, *α*-hydroxyketones; PQS, quinolone signal.


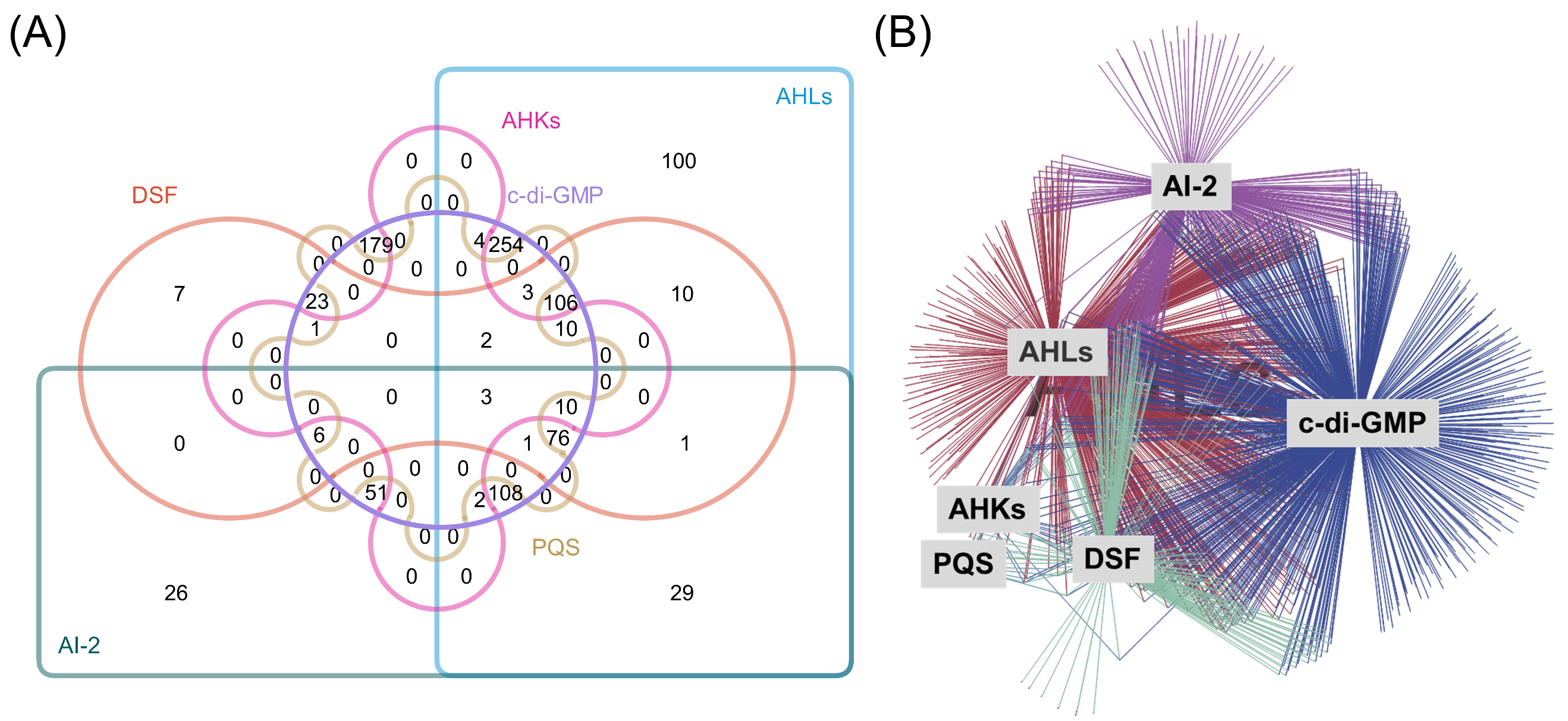


Figure S3 The analysis of QS crosstalk in WWTPs. (A) The distribution of QS bacteria and their specific or shared language system. (B) The connection of different QS languages in AS system. Each node represents a bacterium, and the associated lines indicate that the bacterium has the corresponding language communication system. AS, activated sludge; QS, quorum sensing; QQ, quorum quenching; c-di-GMP, bis-(3’-5’)-cyclic dimeric guanosine monophosphate; AHLs, acyl-homoserine lactones; DSF, diffusion-signaling factors; AI-2, autoinducers-2; AHKs, *α*-hydroxyketones; PQS, quinolone signal.


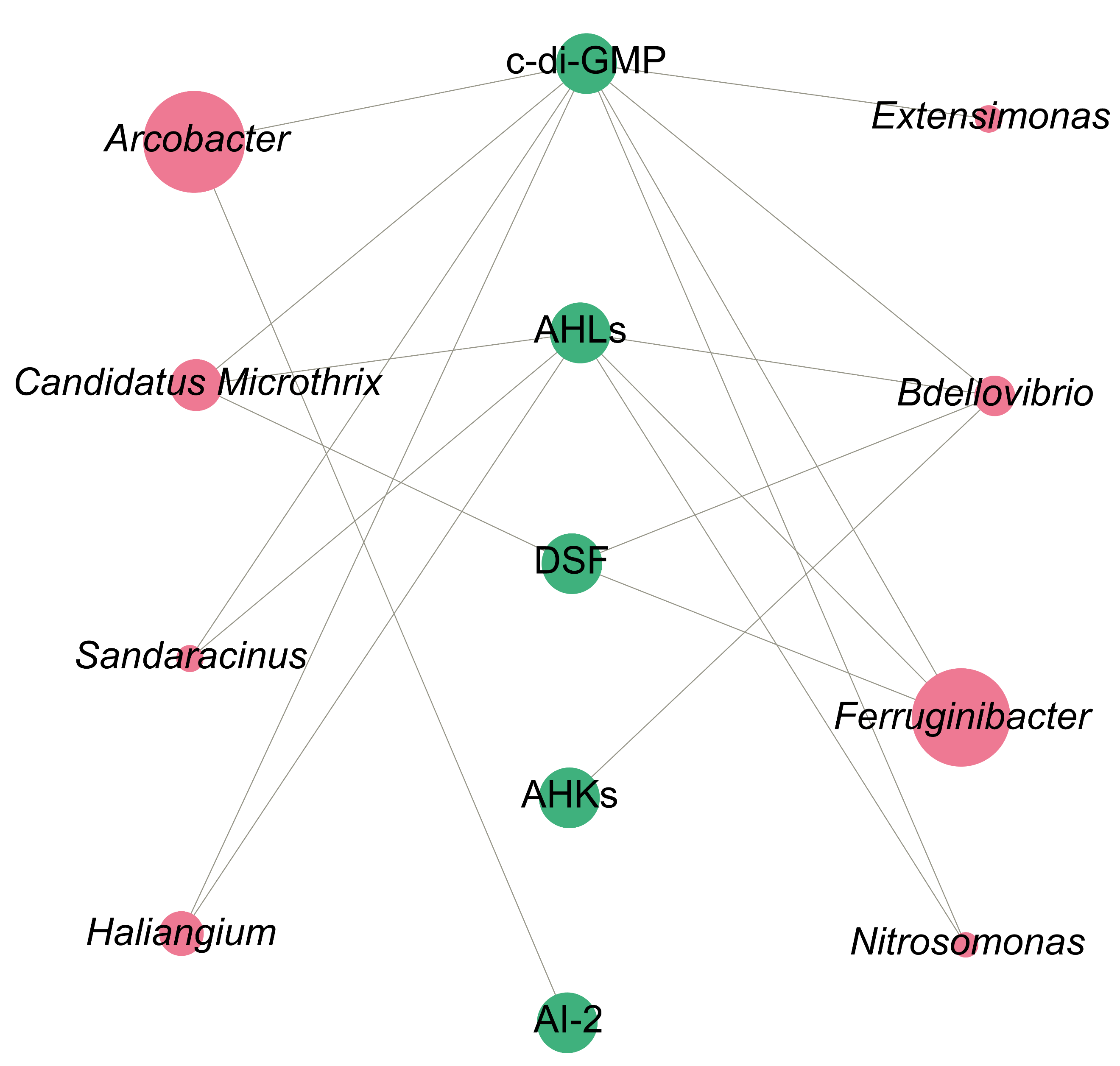


Figure S4 Diagram of potential communication languages between the top 10 genera in relative abundance in WWTPs and the diverse QS languages. c-di-GMP, bis-(3’-5’)-cyclic dimeric guanosine monophosphate; AHLs, acyl-homoserine lactones; DSF, diffusion-signaling factors; AI-2, autoinducers-2; AHKs, α-hydroxyketones; PQS, quinolone signal.


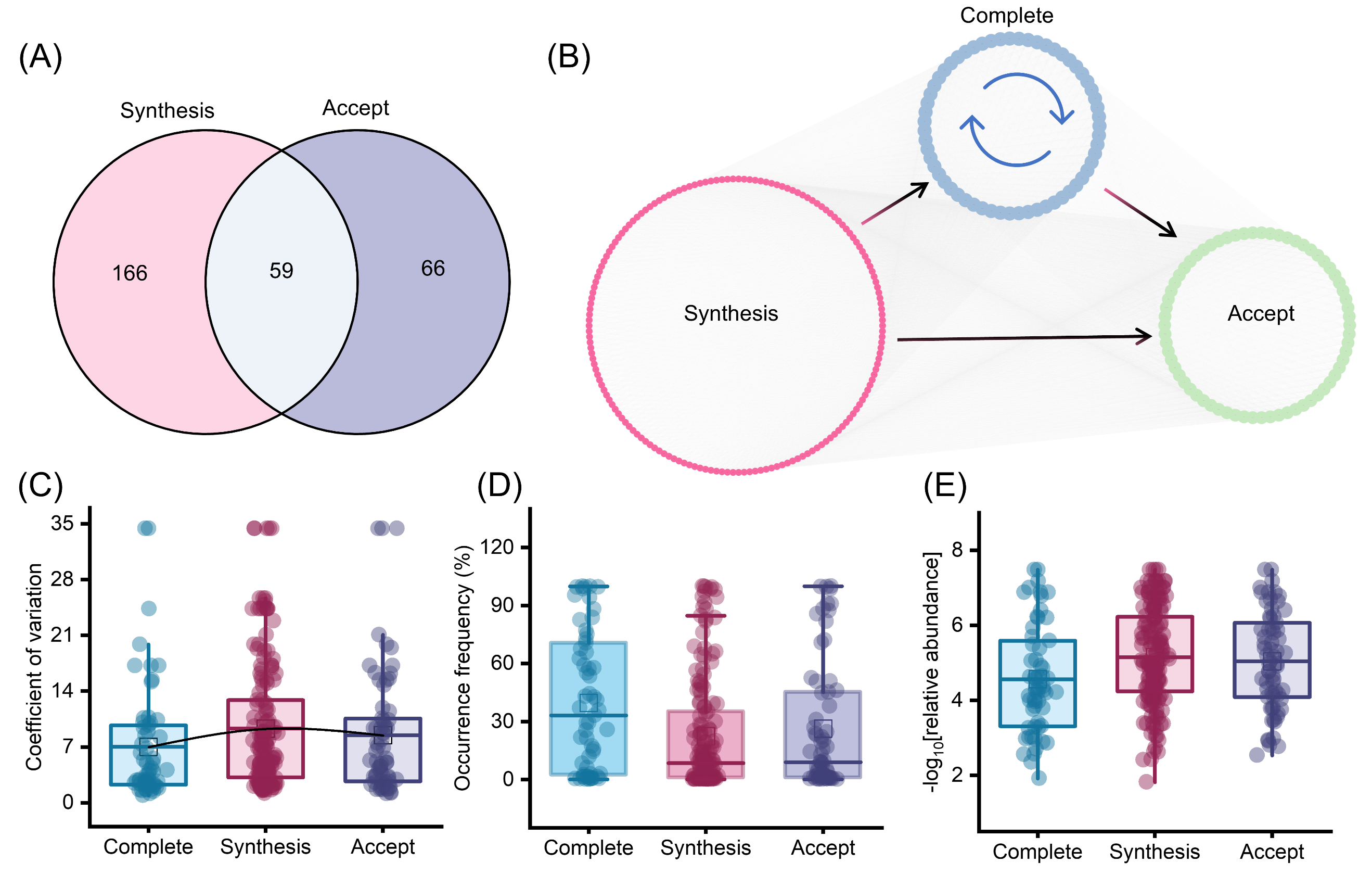
Figure S5 Analysis of the AI-2 type QS bacteria in WWTPs. (A) The Venn diagram shows the number of QS bacteria that contain the function of AI-2 synthesis and acceptance. (B) AI-2-type QS bacteria directed communication network is established from AI-2 signal molecule synthesis to acceptance. The coefficient of variation (C), occurrence frequency (D), and relative abundance (E) analysis show the difference of the three categories of AI-2 related QS bacteria in WWTPs.


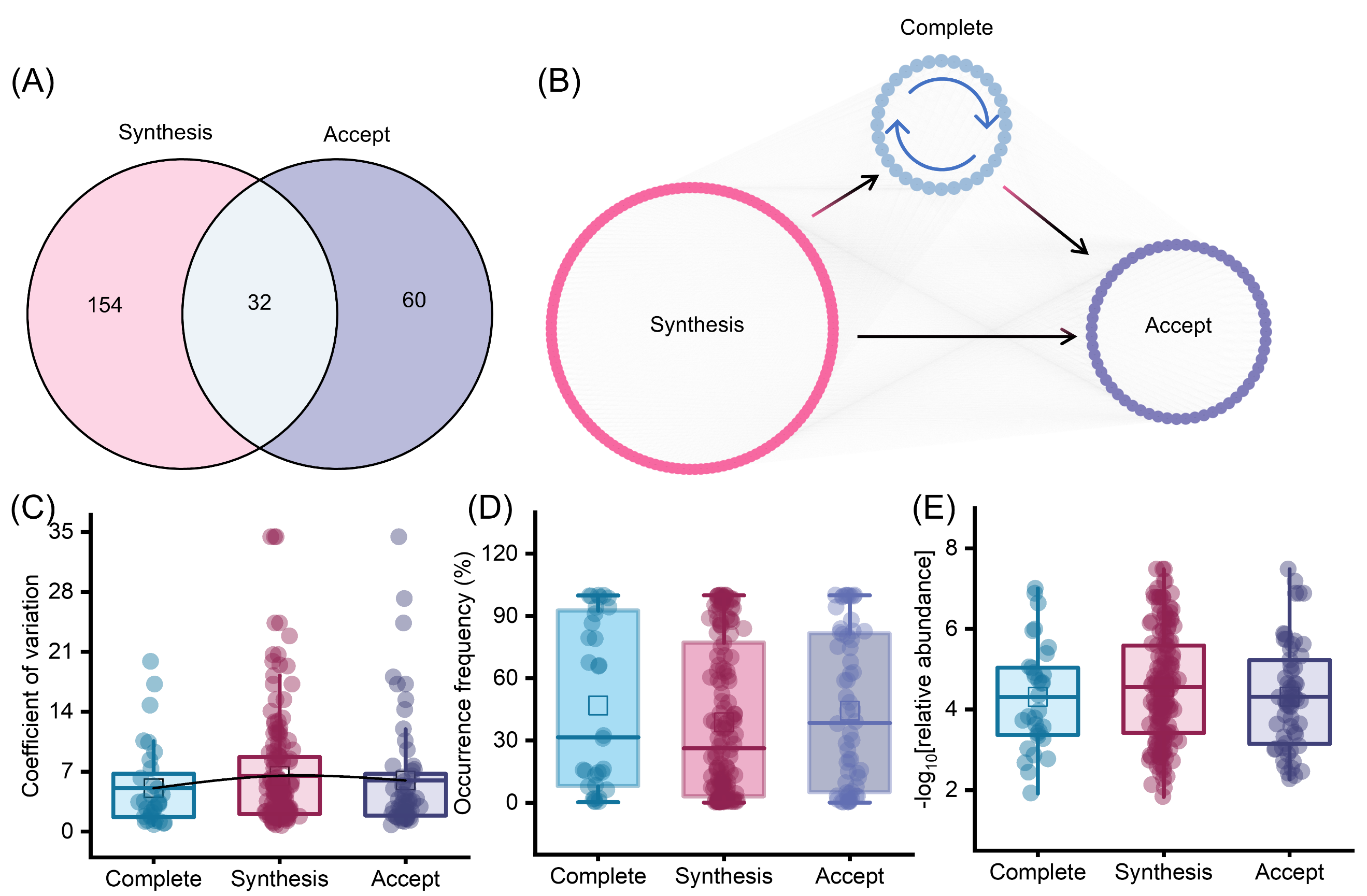


Figure S6 Analysis of the DSF type QS bacteria in WWTPs. (A) The Venn diagram shows the number of QS bacteria that contain the function of DSF synthesis and acceptance. (B) DSF -type QS bacteria directed communication network is established from DSF signal molecule synthesis to acceptance. The coefficient of variation (C), occurrence frequency (D), and relative abundance (E) analysis show the difference of the three categories of DSF related QS bacteria in WWTPs.


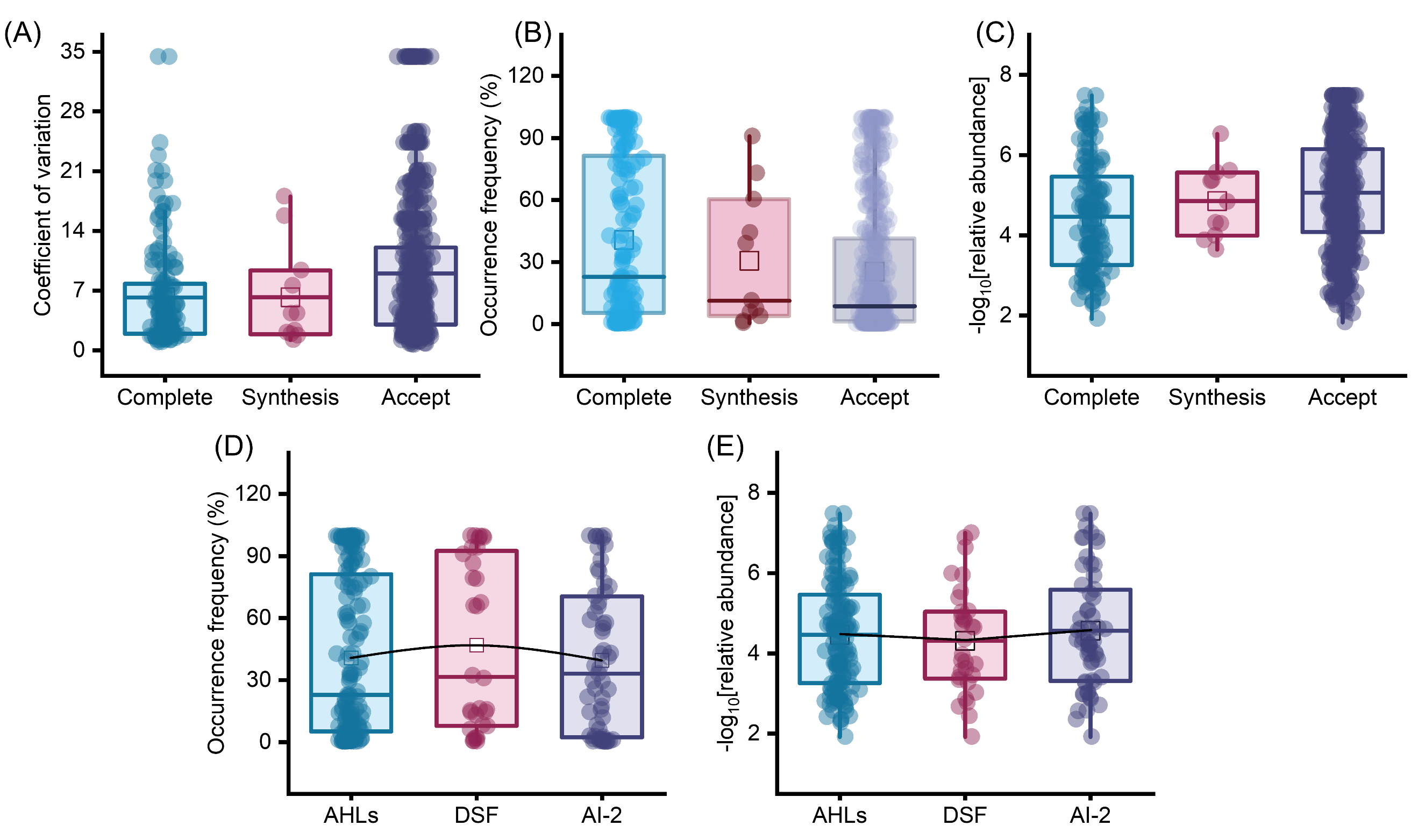


Figure S7 Comparison of various types of QS bacteria in WWTPs. (A) The coefficient of variation, (B) occurrence frequency, and (C) relative abundance analysis show the difference of the three categories of AHL related QS bacteria in WWTPs. (D) occurrence frequency, and (E) relative abundance analysis show the difference between the three types of QS completers in WWTPs. AHLs, acyl-homoserine lactones; DSF, diffusion-signaling factors; AI-2, autoinducers-2.


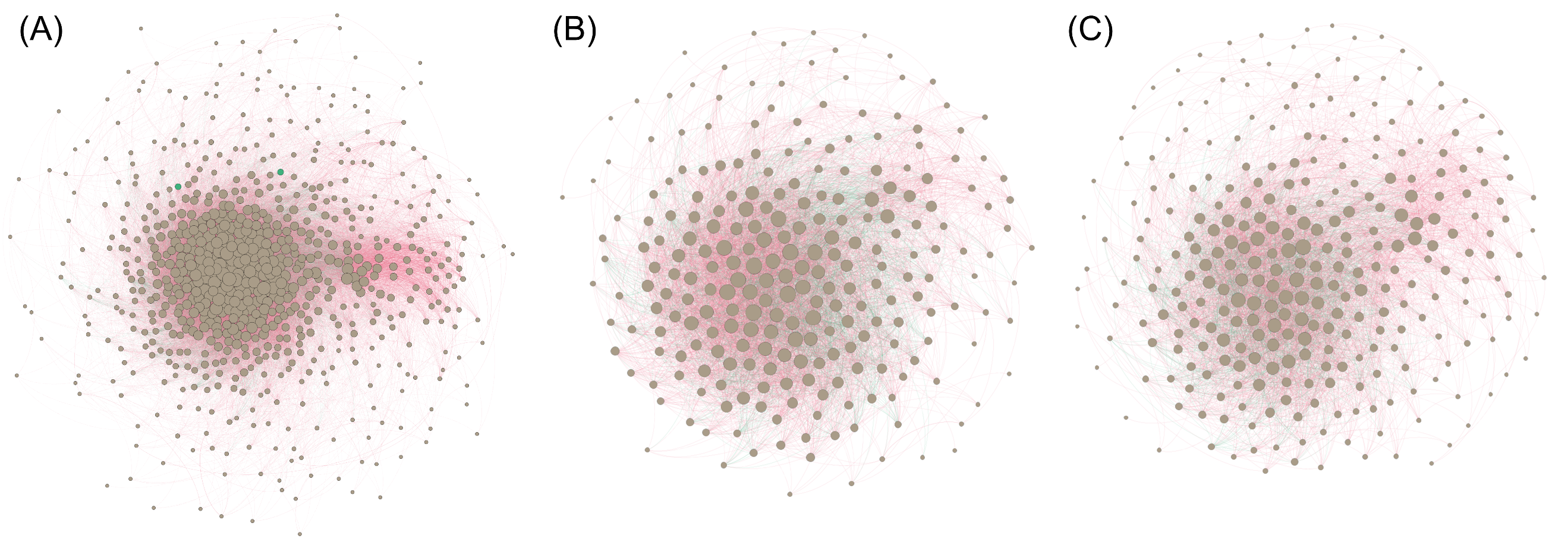


Figure S8 Co-occurrence networks of AHLs (A), DSF (B), and AI-2 (C) types of QS bacteria. The nodes indicated the corresponding bacteria, and the lines indicated the positive (red) and negative (green) correlations of the nodes.


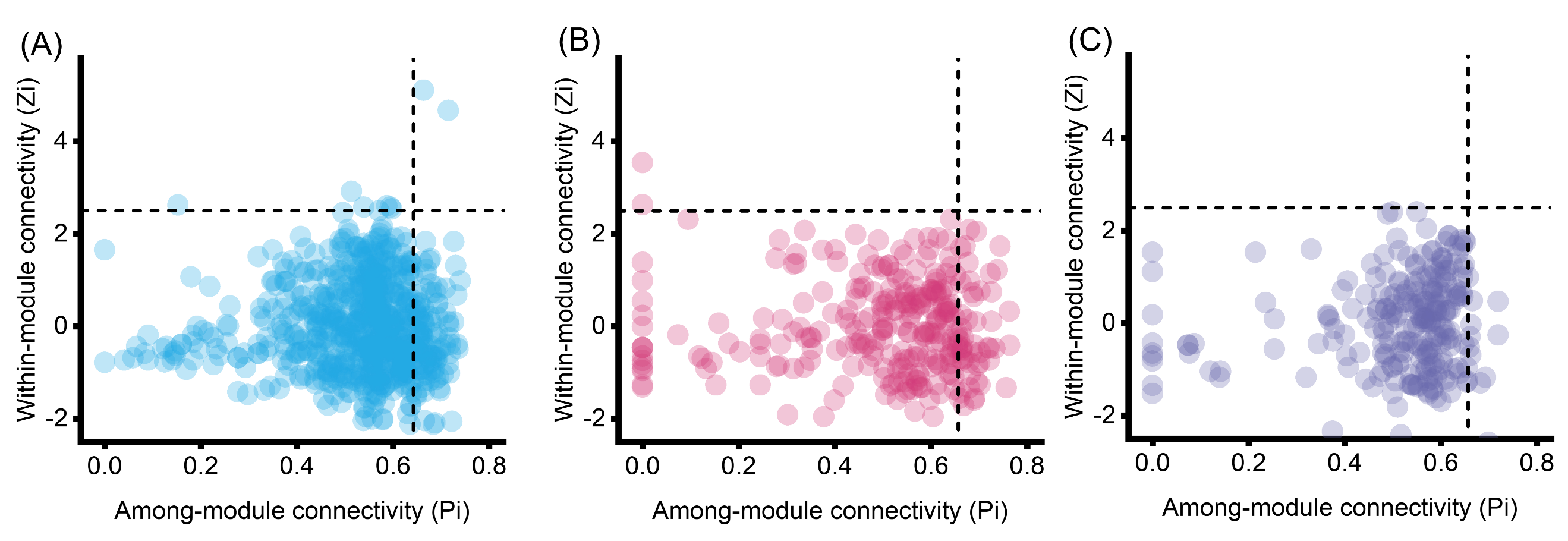


Figure S9 Identification of keystone bacteria in the (A) AHLs, (B) AI-2 and (C) DSF types of QS bacterial co-occurrence networks.


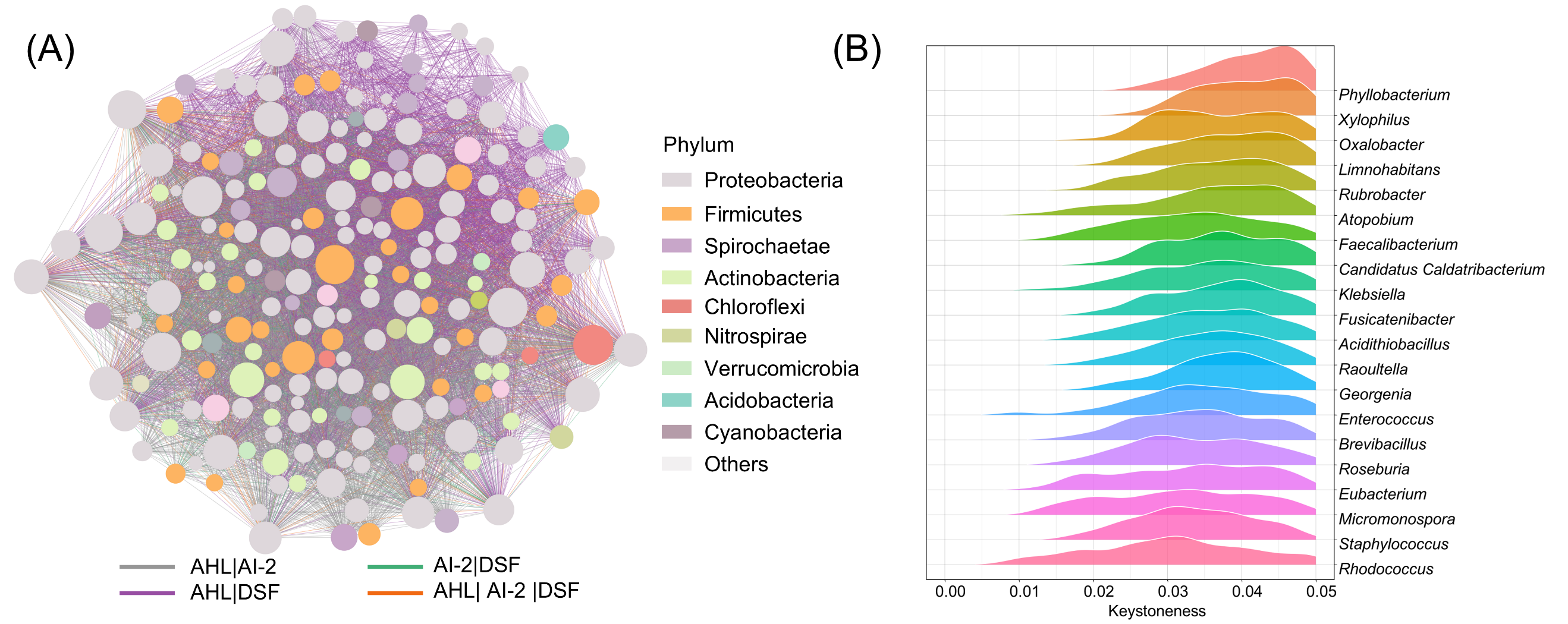


Figure S10 Bacterial interspecific communication network construction. (A) Bacterial interspecific communication network based on AHLs, DSF, and AI-2 types of QS language. The color of the edge indicates the language type, the size of the node indicates the node degree, and the color of the node indicates the bacterial phyla. (B) Top 20 bacteria ranked by their keystoneness in the interspecific communication network. AHLs, acyl-homoserine lactones; DSF, diffusion-signaling factors; AI-2, autoinducers-2.


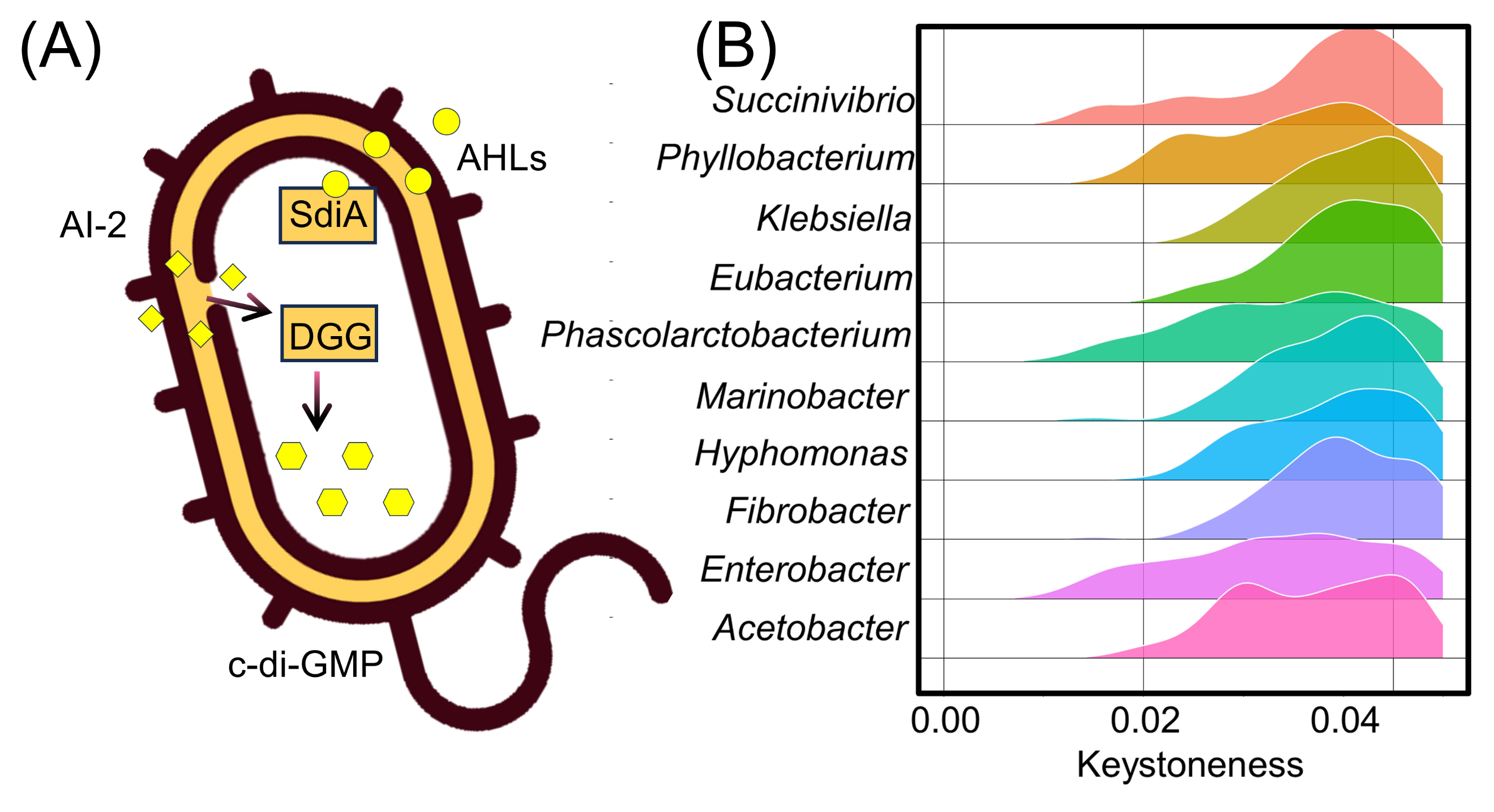


Figure S11 Analysis of intraspecific communication networks in WWTPs. (A) Diagram of the coexistence of multilingual systems within Salmonella inferred from intraspecific bacterial communication networks. These QS signals were verified by previous studies [20,21]. (B) Top 10 bacteria ranked by their keystoneness in the intraspecific communication network. AHLs, acyl-homoserine lactones; c-di-GMP, bis-(3’-5’)-cyclic dimeric guanosine monophosphate; AI-2, autoinducers-2.


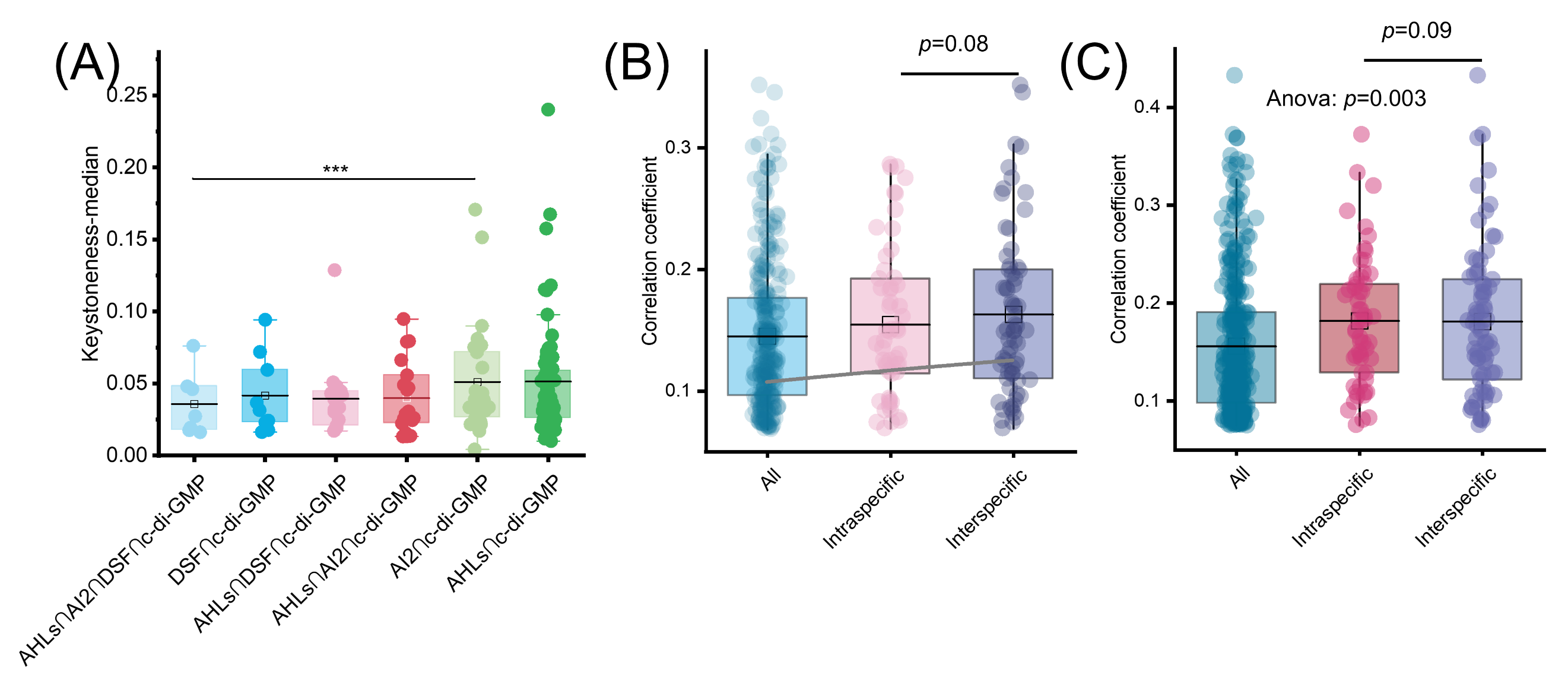


Figure S12 Analysis of various QS bacteria in influencing the functions of WWTPs. (A) Bacteria keystoneness distribution in different multilevel intraspecific language communication systems. (B) Spearman’s rank correlation coefficients of the bacteria abundance in the WWTPs and the BOD removal performance. (C) Spearman’s rank correlation coefficients of the bacteria abundance in the WWTPs and the nitrogen removal performance. The three box plots illustrate the distribution of correlation value of the bacteria that are significantly positively correlated in the entire community, intraspecific QS bacteria, and interspecific QS bacteria. The student’s *t* test was used to analyze the significant difference between the two groups. c-di-GMP, bis-(3’-5’)-cyclic dimeric guanosine monophosphate; AHLs, acyl-homoserine lactones; DSF, diffusion-signaling factors; AI-2, autoinducers-2; AHKs, *α*-hydroxyketones; PQS, quinolone signal.


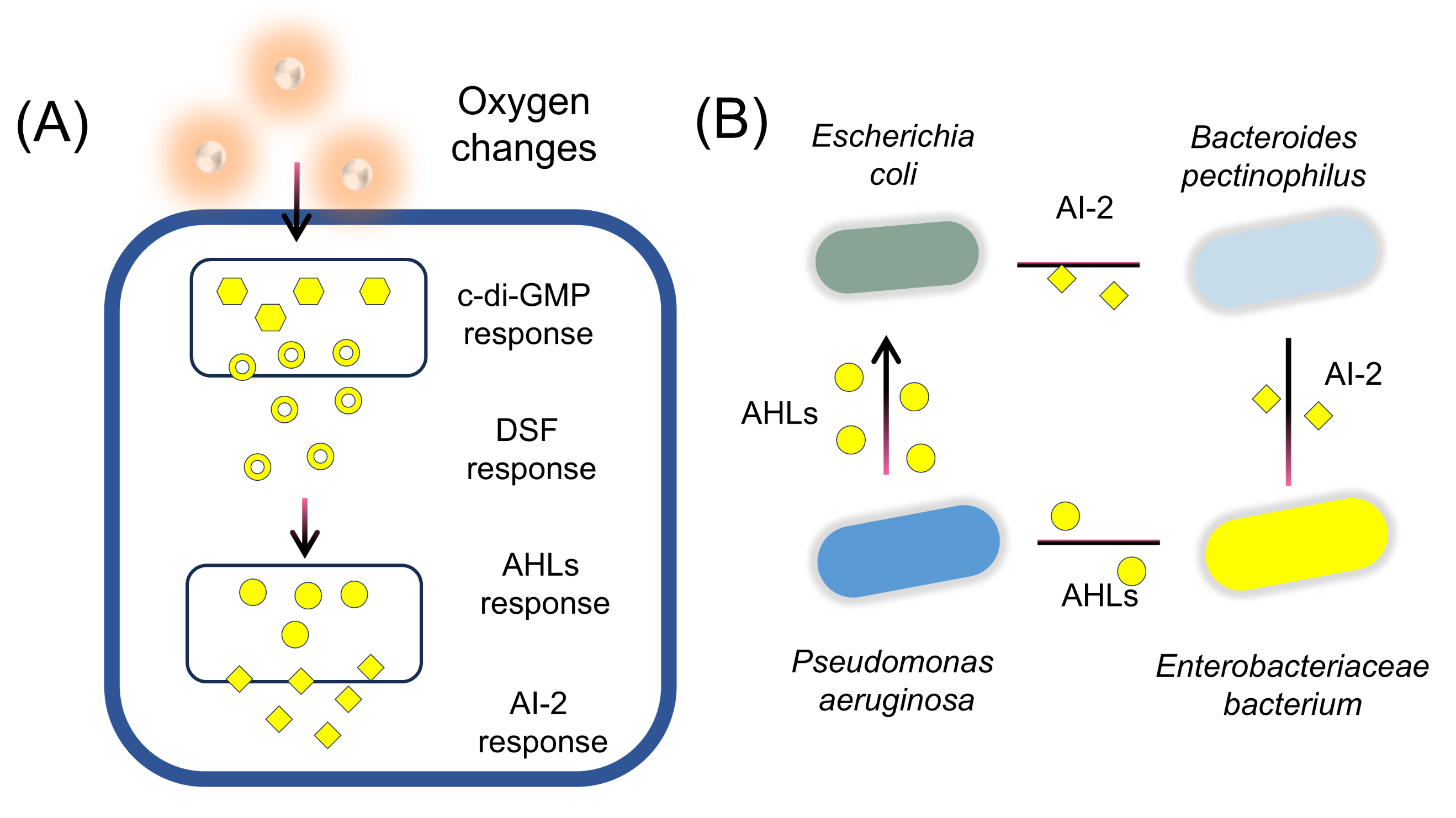


Figure S13 Schematic diagram of microbial QS interspecific communication. (A) Schematic diagram of multiple language systems acting together in response to environmental stress in a microbial community [11]. (B) Schematic diagram of bacterial interspecific communication in the human gut flora [9]. c-di-GMP, bis-(3’-5’)-cyclic dimeric guanosine monophosphate; AHLs, acyl-homoserine lactones; DSF, diffusion-signaling factors; AI-2, autoinducers-2.


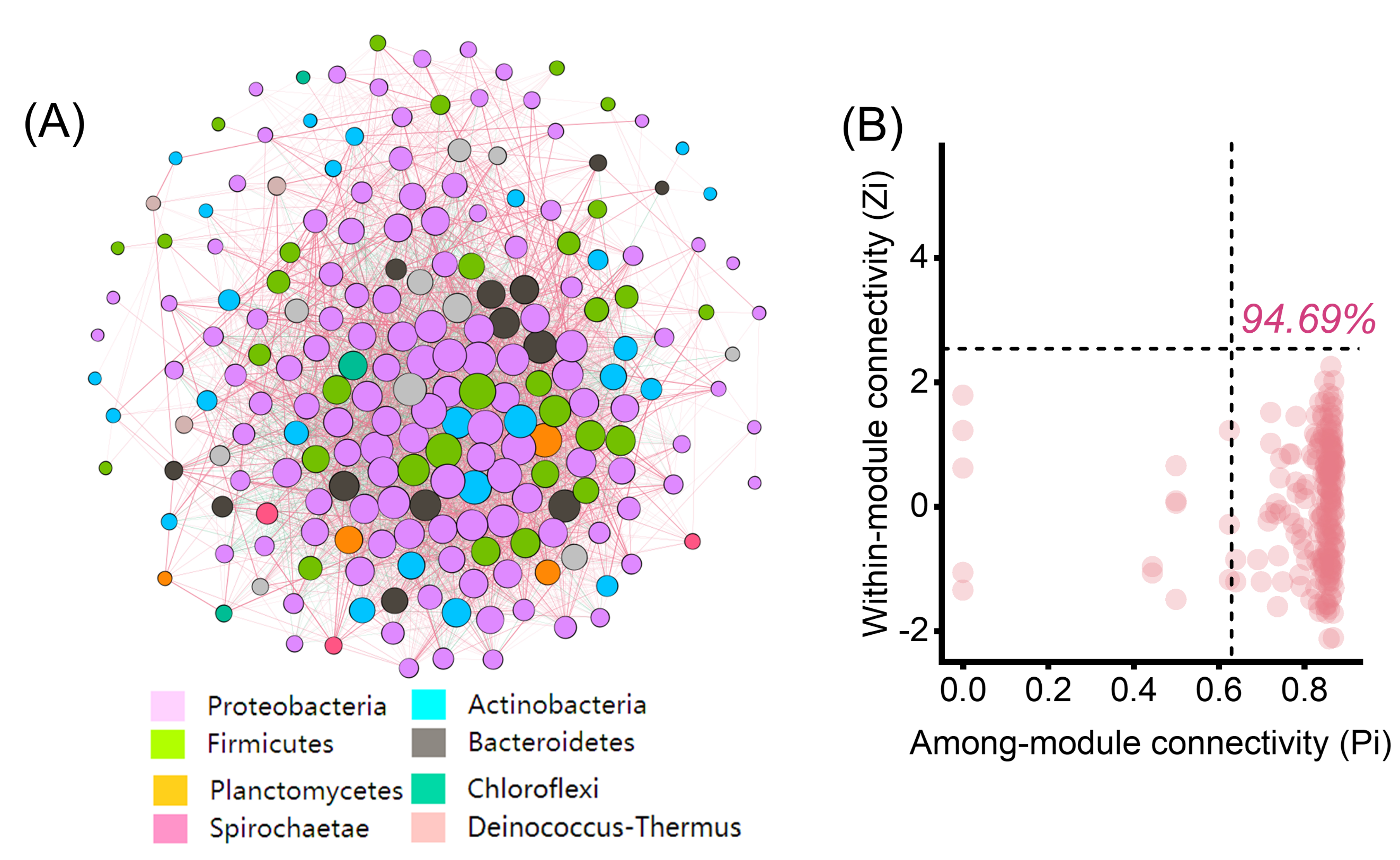


Figure S14 Co-occurrence network of the interspecific QS bacteria. (A) Co-occurrence networks of QS bacteria related to interspecific communication. The nodes indicated the corresponding bacteria, and the lines indicated the positive (red) and negative (green) correlations of the nodes. (B) Identification of keystone bacteria in the interspecific bacterial co-occurrence network.


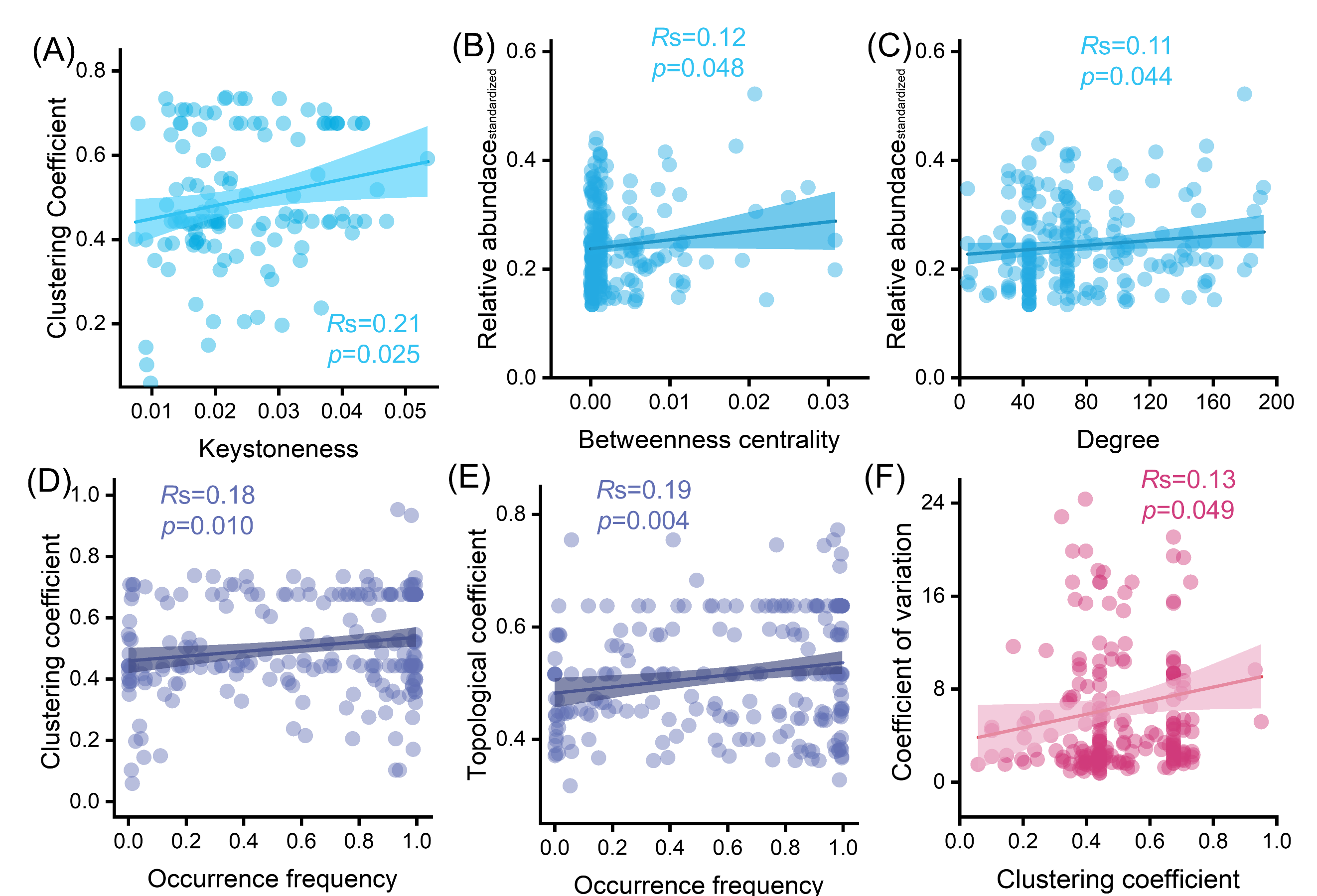


Figure S15 Linear fittings between the parameters between QS communication network and co-occurrence network. (A) Linear fittings between the clustering coefficient of bacteria in QS interspecific communication network and bacterial keystoneness. Linear fittings between the bacterial relative abundance and betweenness centrality (B) and degree (C) of the nodes in the co-occurrence network. Linear fittings between the bacterial occurrence frequency and clustering coefficient (D) and topological coefficient (E) of the nodes in the co-occurrence network. Linear fittings between the bacterial coefficient of variation and clustering coefficient (F) of the nodes in the co-occurrence network.


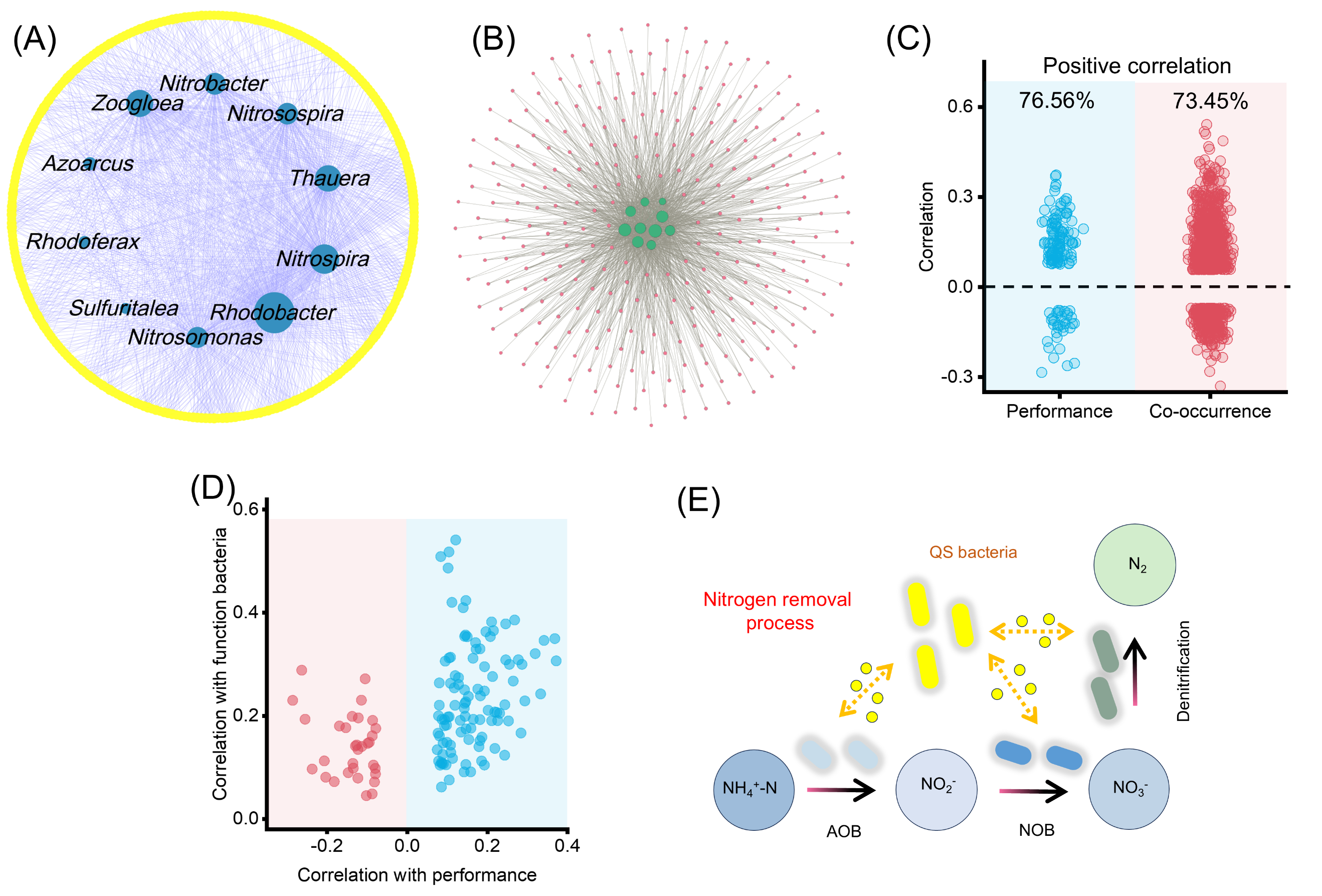


Figure S16 The linkage between bacterial communication and the functions of WWTPs. (A) Bacterial communication relationship of nitrogen removing functional bacteria and QS bacteria in activated sludge community. The edges represent the potential communication links between two nodes. The blue and yellow nodes indicate functional bacteria and other QS bacteria, respectively. (B) Co-occurrence networks of nitrogen removing functional bacteria (green nodes) and linked QS bacteria (red nodes) in activated sludge community. (C) Spearman’s rank correlation coefficients between the abundance of QS bacteria and nitrogen removal performance (blue) and functional bacteria (red) in global WWTPs. The results shown are all significantly correlated (*p* < 0.05). (D) The distribution of Spearman’s rank correlation coefficients for QS bacteria that are significantly correlated with both performance and functional bacteria. (E) Schematic diagram of the influence of QS bacteria on nitrogen degradation in wastewater treatment. AOB, ammonia oxidizing bacteria; NOB, nitrite oxidizing bacteria.


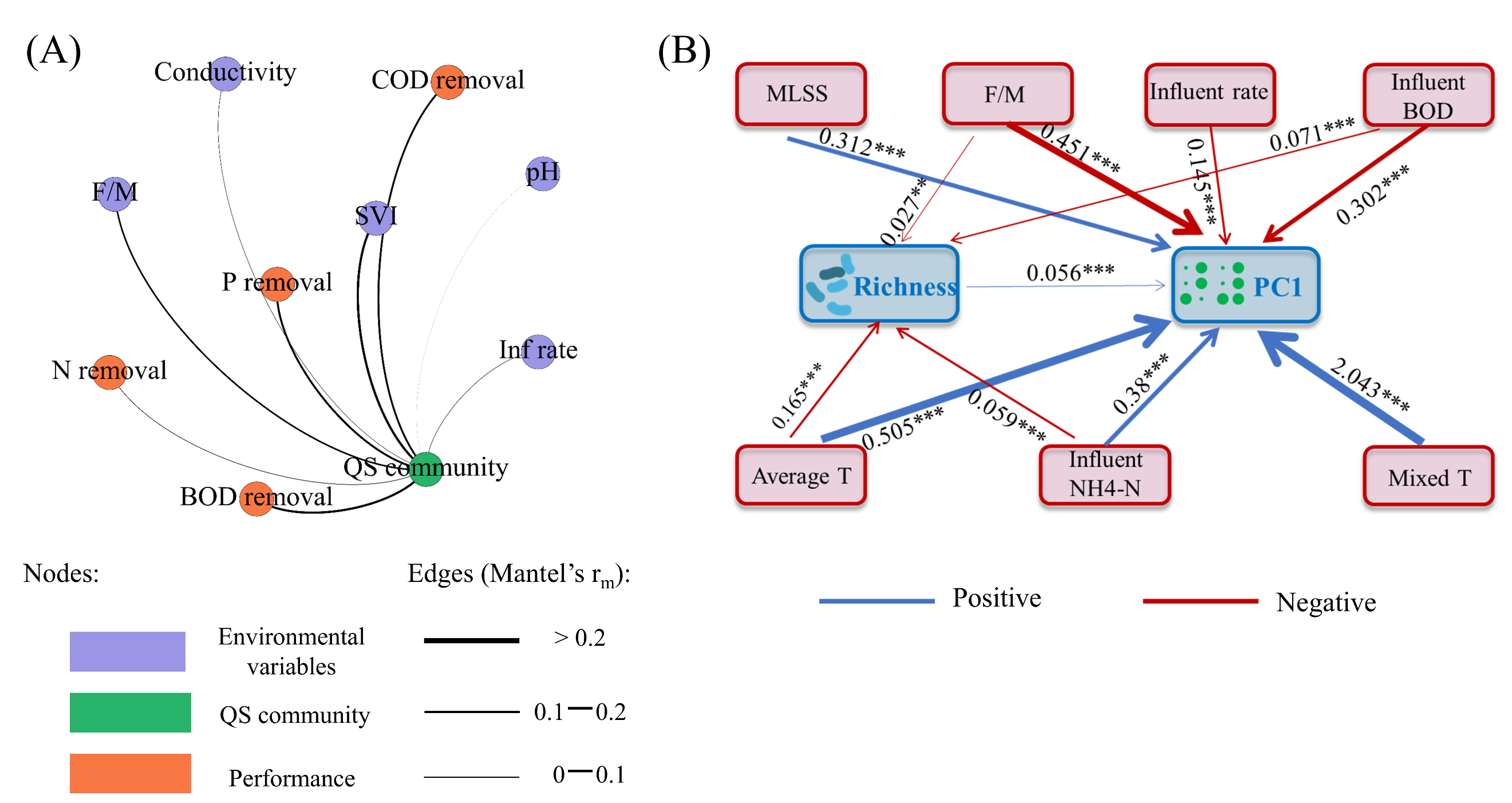


Figure S17 The analysis of the impact of operation conditions on QS community. (A) Mantel tests showed the relationship between bacterial taxonomic community composition of QS bacteria (Bray-Curtis distance) and environmental factors. All of the edges denote the significant correlation (*p* < 0.05) based on 999 permutations and the width of the edges corresponds to Mantel’s r_m_ statistic. (B) The relationships among environmental variables and community composition of QS bacteria are further examined using the SEM model. Richness and PC1 of the community represent the diversity and composition, respectively. All of the edges represented the significant pathways (*p* < 0.05). The number means the standard path coefficients (*β*). The width of the arrow indicates the relationship strength.
